# Supplementary material for: SpaDecon: cell-type deconvolution in spatial transcriptomics with semi-supervised learning
Source: Commun Biol. 2023 Apr 7;6:378. doi: 10.1038/s42003-023-04761-x (PMC10082183; doi:10.1038/s42003-023-04761-x)
Supplement: Supplementary file 2 — Supplementary information [file 42003_2023_4761_MOESM2_ESM.pdf]

Supplementary Information

SpaDecon: cell-type deconvolution in spatial transcriptomics with semi-supervised learning

Kyle Coleman*, Jian Hu, Amelia Schroeder, Edward B. Lee, Mingyao Li*

***Correspondence:**
Kyle Coleman, kylecole@pennmedicine.upenn.edu
Mingyao Li, mingyao@pennmedicine.upenn.edu

Supplementary Table 1. Datasets analyzed in this paper.

Species	Tissue	Data Source	Dataset Dimensions	Protocol
Mouse	Anterior brain (sagittal)	10X Genomics https://support.10xgenomics.com/spatial-gene-expression/datasets/1.1.0/V1_Mouse_Brain_Sagittal_Anterior	2,695 spots<br>32,285 genes	10x Visium
Mouse	Whole cortex and hippocampus	Yao *et al*. [1] https://portal.brain-map.org/atlases-and-data/rnaseq/mouse-whole-cortex-and-hippocampus-smart-seq	<u>Original:</u><br>76,533 cells<br>45,768 genes<br><br><u>Reduced:</u><br>2,000 cells<br>45,768 genes	SMART-seq
Human	Invasive Ductal Carcinoma breast tissue	10X Genomics https://support.10xgenomics.com/spatial-gene-expression/datasets/1.1.0/V1_Breast_Cancer_Block_A_Section_1	3,798 spots<br>36,601 genes	10x Visium
Human	Breast cancer tissue	Wu *et al*. [2]<br>GSE176078	<u>Original:</u><br>100,064 cells<br>29,733 genes<br><br><u>Reduced:</u><br>2,000 cells<br>29,733 genes	10x Chromium
Human	Stage III cutaneous malignant melanoma tissue	Thrane *et al*. [3] https://www.spatialresearch.org/resources-published-datasets/doi-10-1158-0008-5472-can-18-0747/	293 spots<br>16,148 genes	Spatial Transcriptomics
Human	Metastatic melanoma tissue	Tirosh *et al*. [4]<br>GSE72056	4,139 cells<br>23,686 genes	Smart-seq2
Human	Pancreatic adenocarcinoma tissue	Moncada *et al*. [5]<br>GSM3405534	224 spots<br>19,738 genes	Spatial Transcriptomics
Human	Pancreatic adenocarcinoma tissue	Moncada *et al*. [5]<br>GSE111672	1,733 cells<br>19,738 genes	inDrop

Supplementary Table 2. Software compared with SpaDecon.

Method	Version	URL	Reference
RCTD	1.2.0	https://github.com/dmcable/RCTD	[6]
SPOTlight	0.1.7	https://github.com/MarcElosua/SPOTlight	[7]
Stereoscope	0.2.0	https://github.com/almaan/stereoscope	[8]
cell2location	0.1.0	https://github.com/BayraktarLab/cell2location	[9]
MuSiC	0.2.0	https://github.com/xuranw/MuSiC	[10]

Supplementary Table 3. Cell type abbreviations.

Abbreviation	Full Name
Astro	Astrocytes
CAFs	Cancer-associated fibroblasts
Endo	Endothelial cells
Macro	Macrophages
mDCs	Myeloid dendritic cells
Micro	Microglia
NK	Natural killer cells
Oligo	Oligodendrocytes
Pvalb	Parvalbumin
PVL	Perivascular-like cells
RBCs	Red blood cells
Sst	Somatostatin-expressing neurons
Vip.Sncg	Vasoactive intestinal polypeptide/ synuclein-gamma expressing neurons

Supplementary Figure 1. For each method, heatmaps displaying the estimated distributions of cell types across the 10X Visium mouse anterior brain dataset. Each spot is colored according to the proportion of a given cell type in that spot as estimated by a given method. See Supplementary Table 3 for full names of cell types.

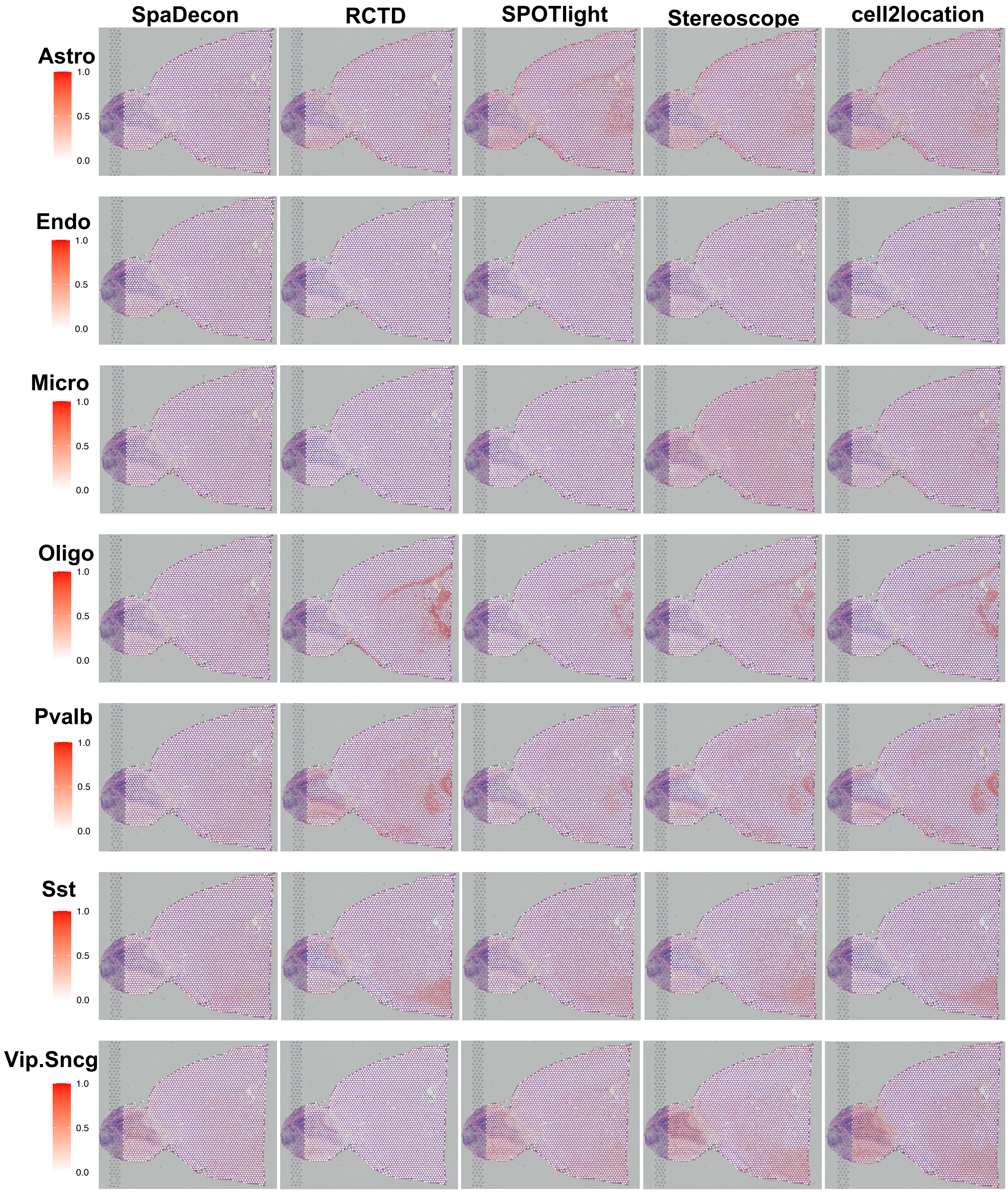

Supplementary Figure 2. For each method, heatmaps displaying the Jensen-Shannon Divergence (JSD) between the proportions estimated using the first scRNA-seq subset as reference and those estimated using each following scRNA-seq subset as reference for cell-type deconvolution of the 10X Visium mouse anterior brain dataset.

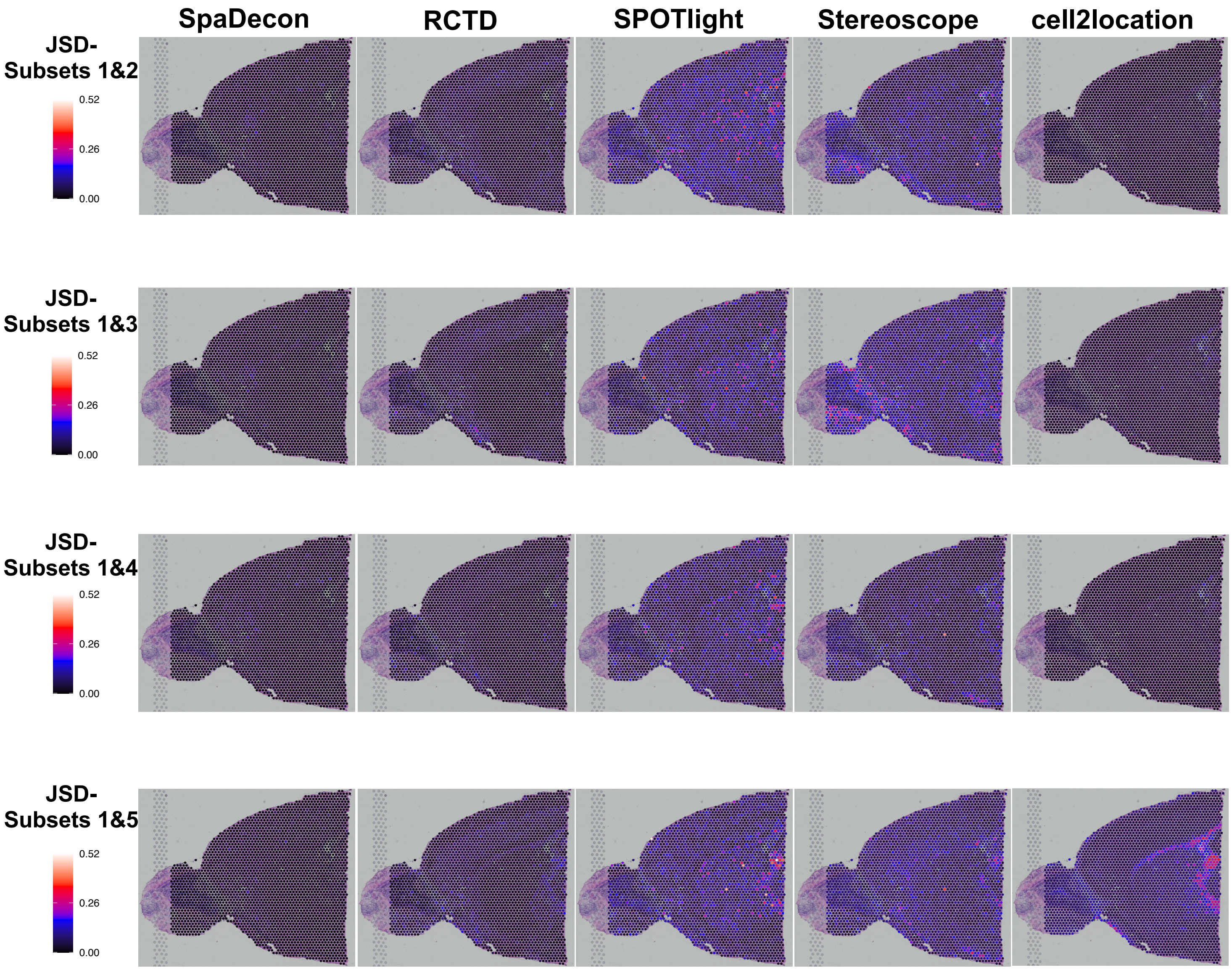

Supplementary Figure 3. 10X Visium mouse anterior brain benchmark evaluations. a, Boxplot showing the mean squared error between the benchmark and estimated proportions across all cell types for each method (n=11 cell types). **b,** Boxplot showing the Jensen-Shannon divergence between the benchmark and estimated proportions across all spots for each method (n=2695 spots). **c,** Heatmaps showing the Jensen-Shannon divergence between the benchmark and estimated proportions at each spot in the pseudo-SRT dataset for each method.

a

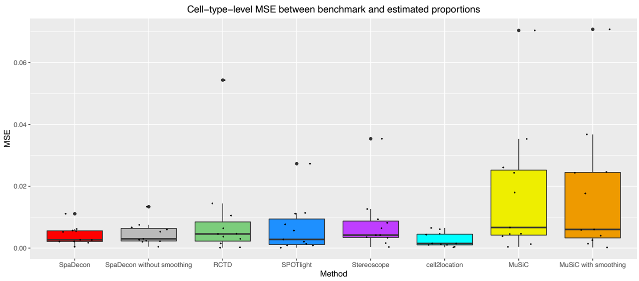

b

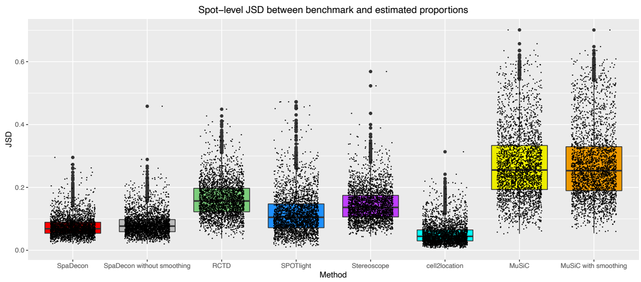

c

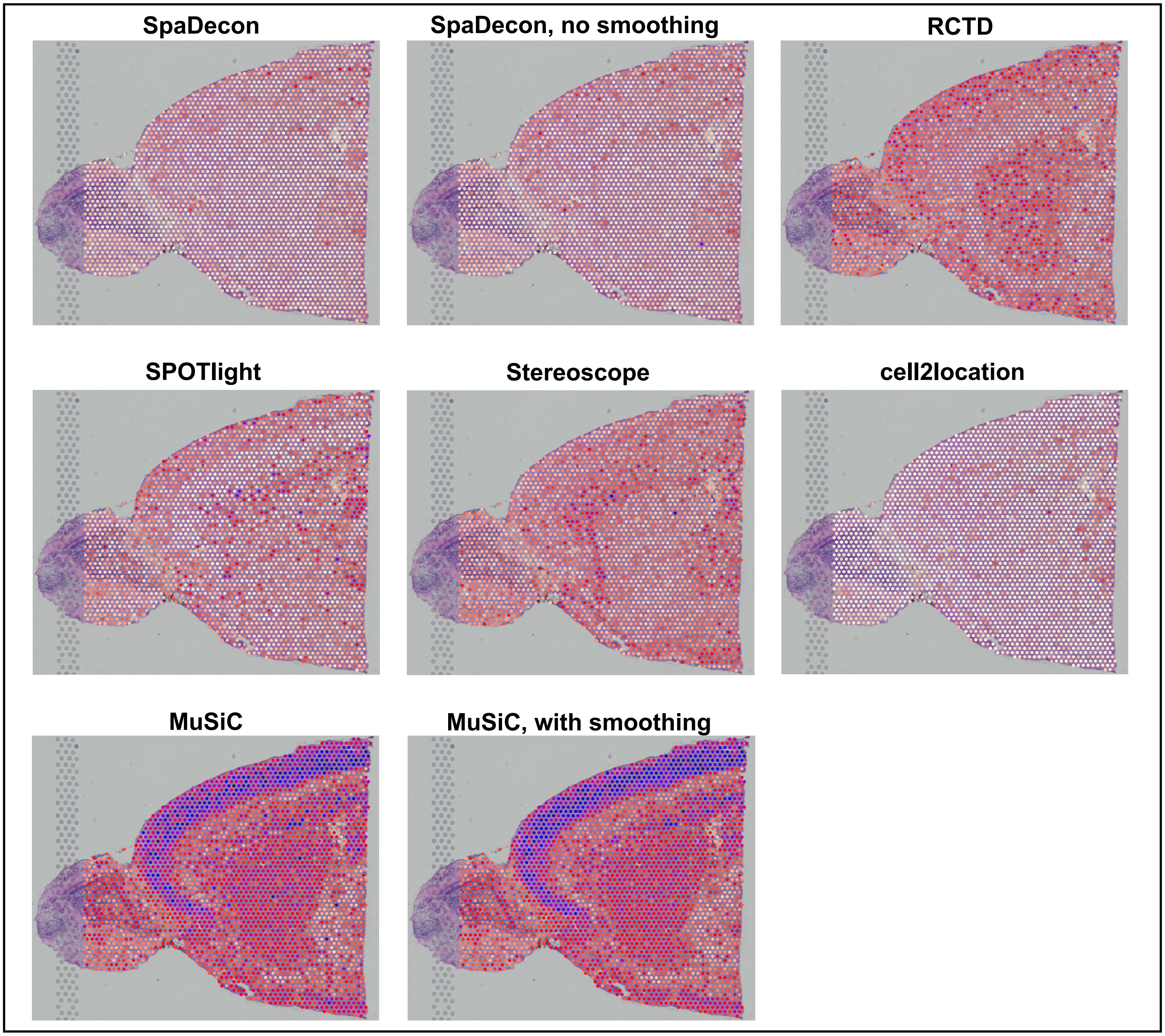

Supplementary Figure 4. For each method, heatmaps displaying the estimated distributions of cell types across the 10X Visium breast cancer dataset. Each spot is colored according to the proportion of a given cell type in that spot as estimated by a given method. See Supplementary Table 3 for full names of cell types.

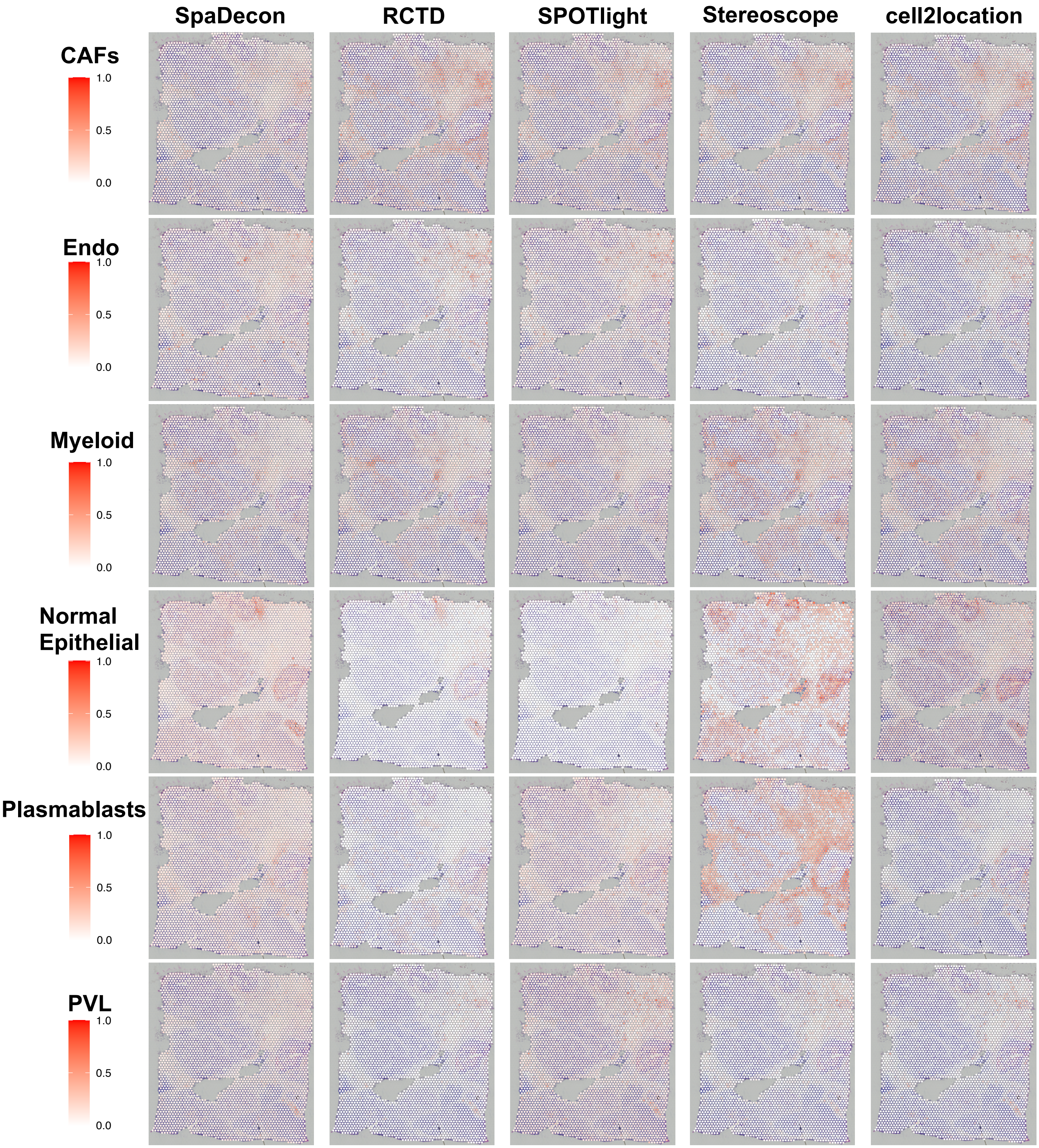

Supplementary Figure 5. For each method, heatmaps displaying the Jensen-Shannon Divergence (JSD) between the proportions estimated using the first scRNA-seq subset as reference and those estimated using each following scRNA-seq subset as reference for cell-type deconvolution of the 10X Visium breast cancer dataset.

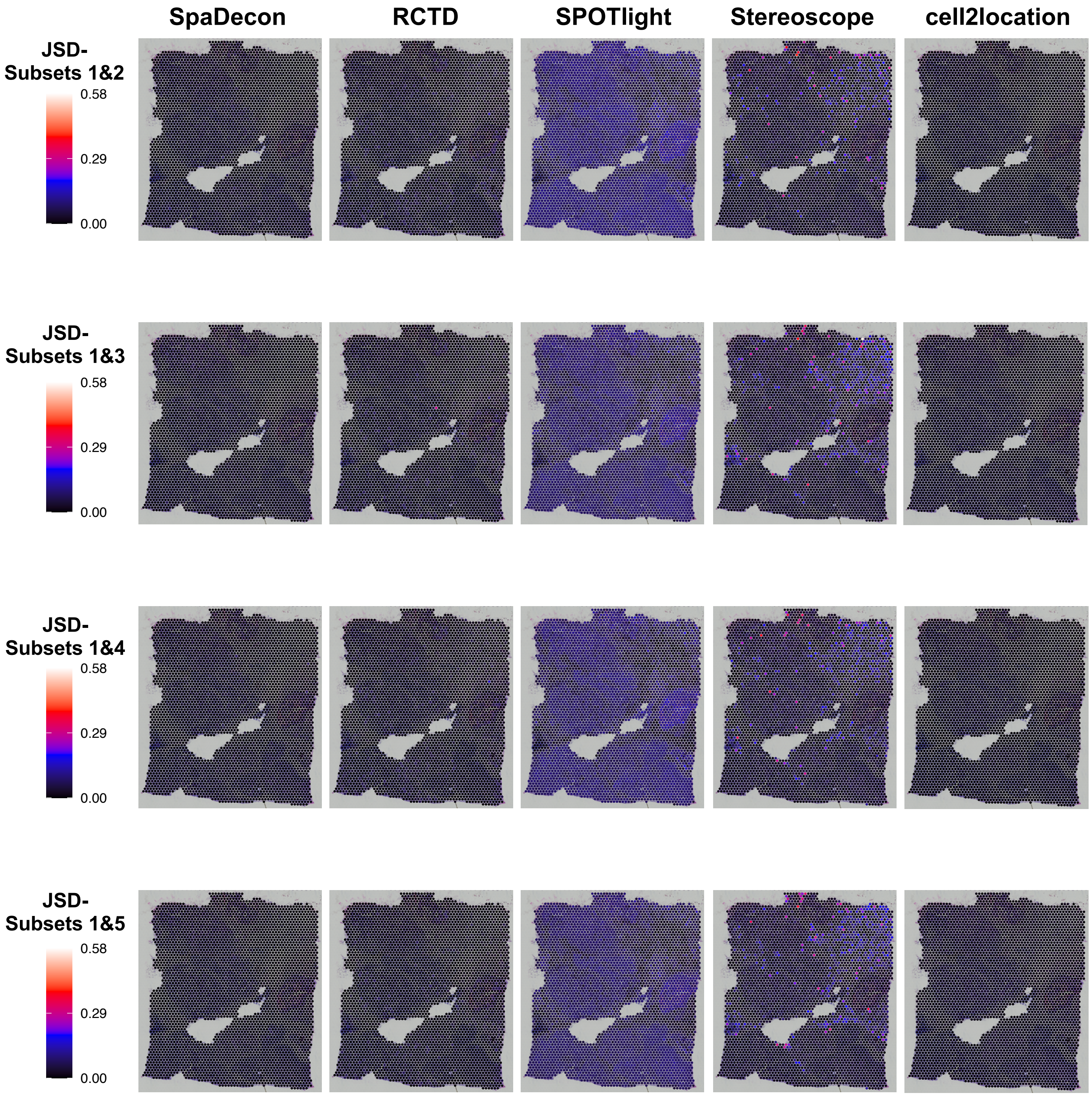

Supplementary Figure 6. 10X Visium breast cancer benchmark evaluations. a, Boxplot showing the mean squared error between the benchmark and estimated proportions across all cell types for each method (n=9 cell types). **b,** Boxplot showing the Jensen-Shannon divergence between the benchmark and estimated proportions across all spots for each method (n=3798 spots). **c,** Heatmaps showing the Jensen-Shannon divergence between the benchmark and estimated proportions at each spot in the pseudo-SRT dataset for each method.

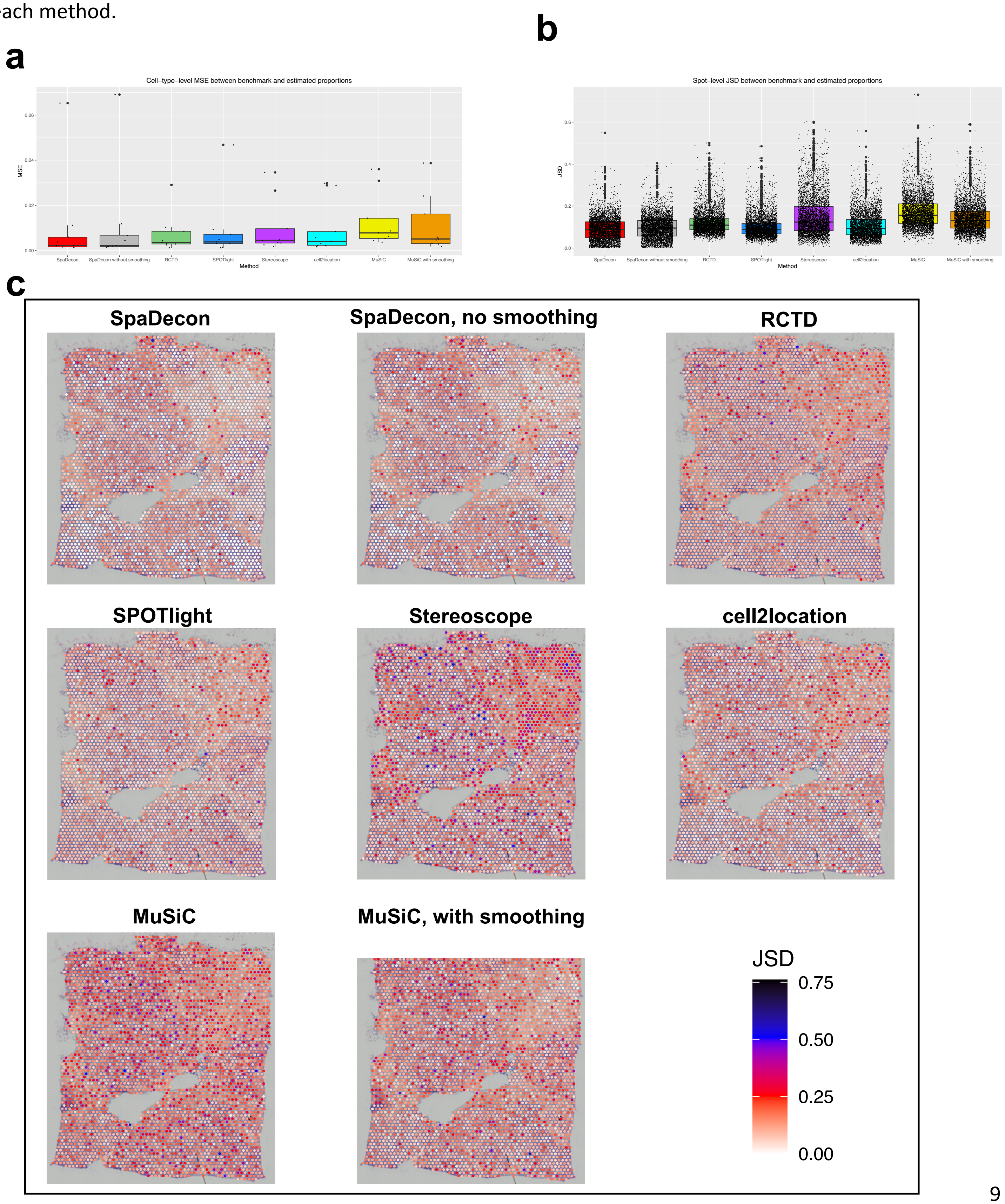

Supplementary Figure 7. For each method, heatmaps displaying the estimated distributions of cell types across the stage III cutaneous malignant melanoma ST dataset. Each spot is colored according to the proportion of a given cell type in that spot as estimated by a given method. See Supplementary Table 3 for full names of cell types.

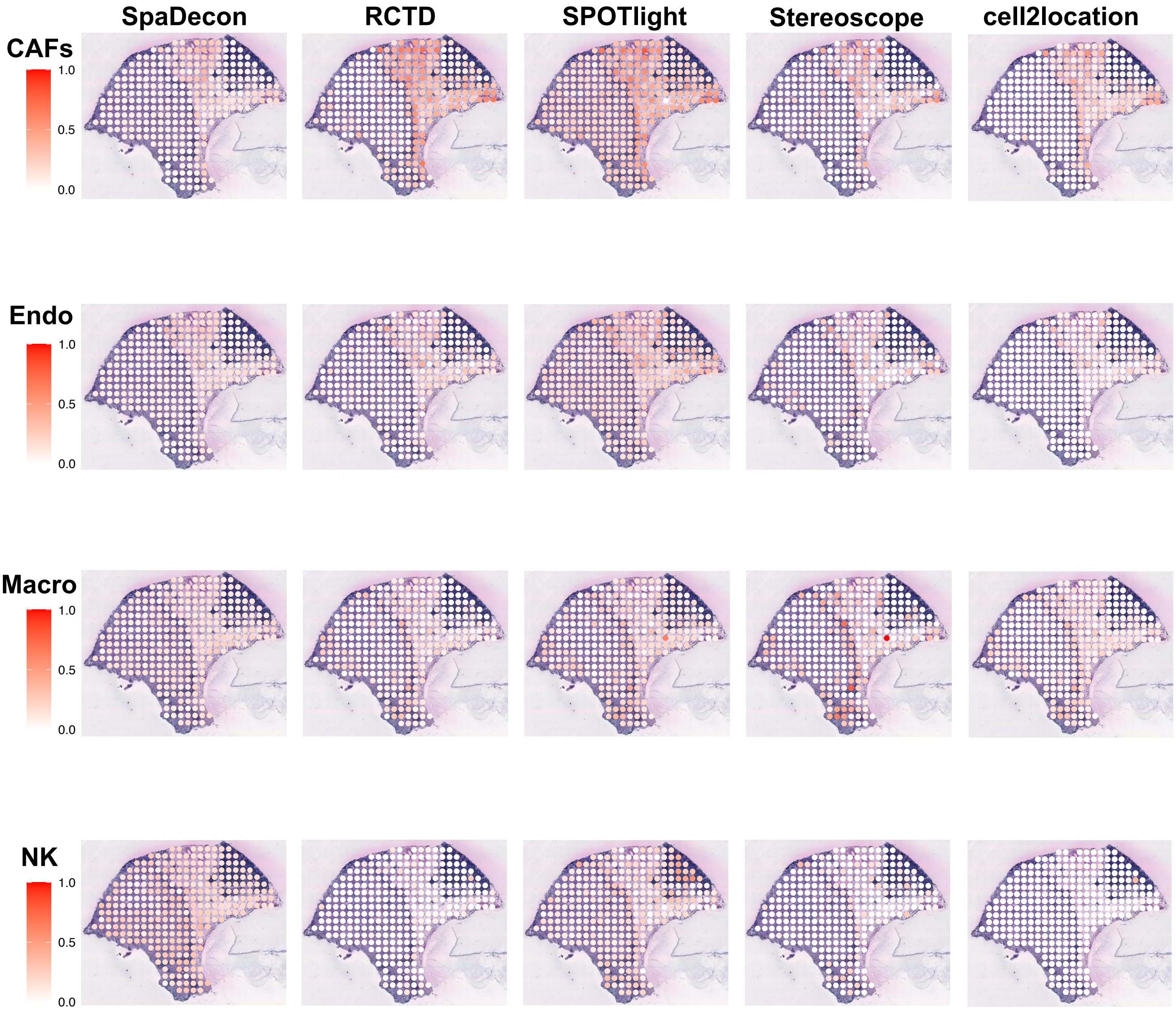

Supplementary Figure 8. Stage III cutaneous malignant melanoma ST benchmark evaluations. a, Boxplot showing the mean squared error between the benchmark and estimated proportions across all cell types for each method (n=7 cell types). **b,** Boxplot showing the Jensen-Shannon divergence between the benchmark and estimated proportions across all spots for each method (n=281 spots). **c,** Heatmaps showing the Jensen-Shannon divergence between the benchmark and estimated proportions at each spot in the pseudo-SRT [illegible]et for each method.

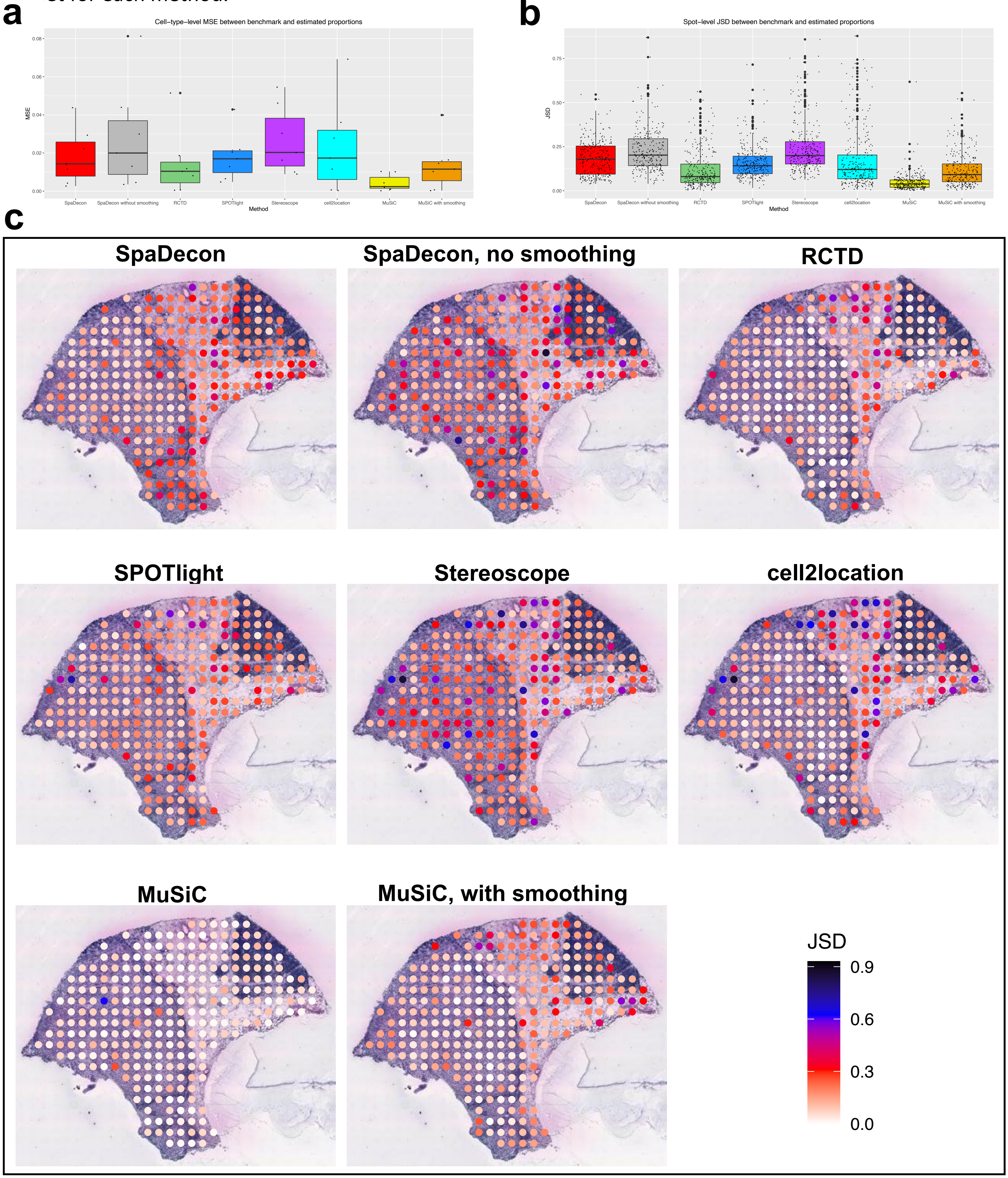

Supplementary Figure 9. For each method, heatmaps displaying the estimated distributions of cell types across the pancreatic ductal adenocarcinoma ST dataset. Each spot is colored according to the proportion of a given cell type in that spot as estimated by a given method. See Supplementary Table 3 for full names of cell types.

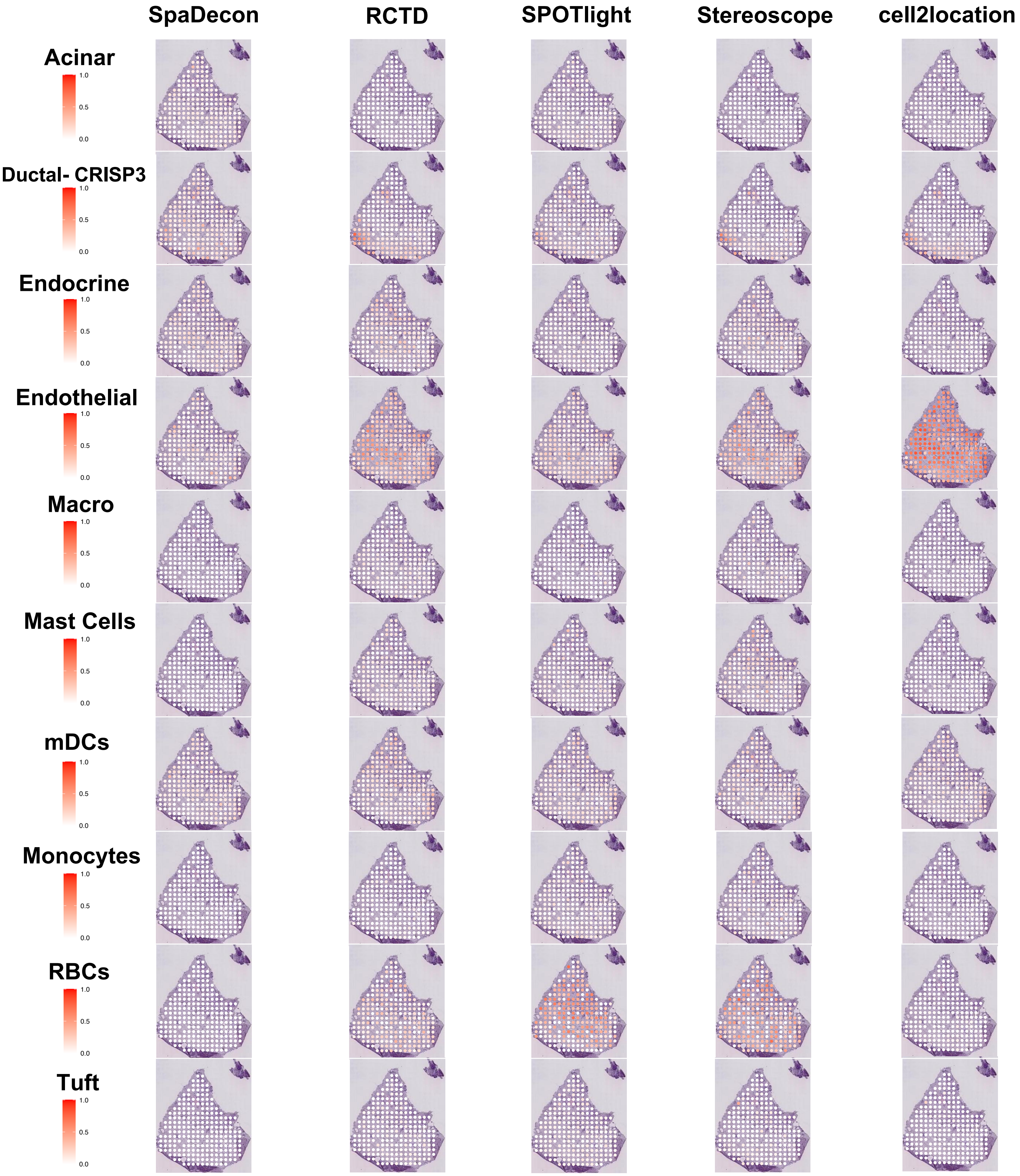

Supplementary Figure 10. Plot detailing the memory usage of SpaDecon when analyzing the 10X Visium breast cancer dataset. Memory was measured every 0.1 seconds and the maximum memory usage was approximately 5.06 gigabytes.

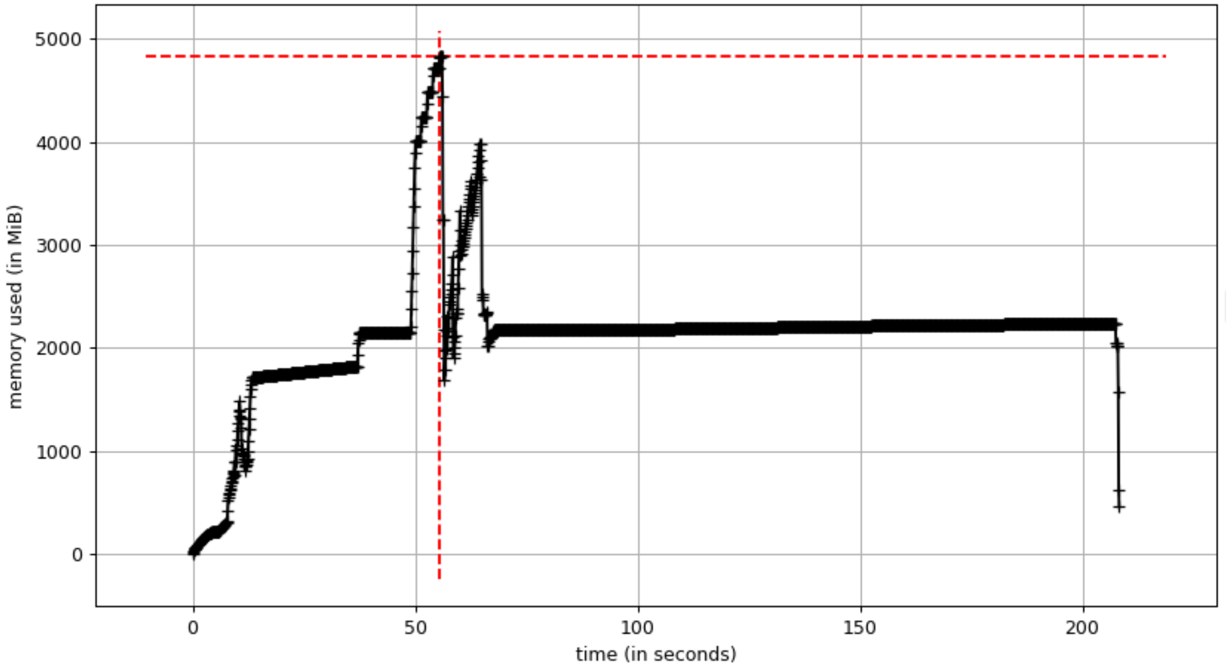

References

[1] Yao Z, van Velthoven CTJ, Nguyen TN, Goldy J, Sedeno-Cortes AE, Baftizadeh F, Bertagnolli D, Casper T, Chiang M, Crichton K, et al: **A taxonomy of transcriptomic cell types across the isocortex and hippocampal formation.** *Cell* 2021, **184:**3222-3241 e3226.

[2] Wu SZ, Al-Eryani G, Roden DL, Junankar S, Harvey K, Andersson A, Thennavan A, Wang C, Torpy JR, Bartonicek N, et al: **A single-cell and spatially resolved atlas of human breast cancers.** *Nat Genet* 2021, **53:**1334-1347.

[3] Thrane K, Eriksson H, Maaskola J, Hansson J, Lundeberg J: **Spatially Resolved Transcriptomics Enables Dissection of Genetic Heterogeneity in Stage III Cutaneous Malignant Melanoma.** *Cancer Res* 2018, **78:**5970-5979.

[4] Tirosh I, Izar B, Prakadan SM, Wadsworth MH, 2nd, Treacy D, Trombetta JJ, Rotem A, Rodman C, Lian C, Murphy G, et al: **Dissecting the multicellular ecosystem of metastatic melanoma by single-cell RNA-seq.** *Science* 2016, **352:**189-196.

[5] Moncada R, Barkley D, Wagner F, Chiodin M, Devlin JC, Baron M, Hajdu CH, Simeone DM, Yanai I: **Integrating microarray-based spatial transcriptomics and single-cell RNA-seq reveals tissue architecture in pancreatic ductal adenocarcinomas.** *Nat Biotechnol* 2020, **38:**333-342.

[6] Cable DM, Murray E, Zou LS, Goeva A, Macosko EZ, Chen F, Irizarry RA: **Robust decomposition of cell type mixtures in spatial transcriptomics.** *Nat Biotechnol* 2021.

[7] Elosua-Bayes M, Nieto P, Mereu E, Gut I, Heyn H: **SPOTlight: seeded NMF regression to deconvolute spatial transcriptomics spots with single-cell transcriptomes.** *Nucleic Acids Res* 2021, **49:**e50.

[8] Andersson A, Bergenstrahle J, Asp M, Bergenstrahle L, Jurek A, Fernandez Navarro J, Lundeberg J: **Single-cell and spatial transcriptomics enables probabilistic inference of cell type topography.** *Commun Biol* 2020, **3:**565.

[9] Kleshchevnikov V, Shmatko A, Dann E, Aivazidis A, King HW, Li T, Elmentaite R, Lomakin A, Kedlian V, Gayoso A, et al**: Cell2location maps fine-grained cell types in spatial transcriptomics.** *Nat Biotechnol* 2022, **40:**661-671.

[10] Wang X, Park J, Susztak K, Zhang NR, Li M: **Bulk tissue cell type deconvolution with multi-subject single-cell expression reference.** *Nat Commun* 2019, **10:**380.
