# Supplementary material for: SpaDecon: cell-type deconvolution in spatial transcriptomics with semi-supervised learning
Source: Commun Biol. 2023 Apr 7;6:378. doi: 10.1038/s42003-023-04761-x (PMC10082183; doi:10.1038/s42003-023-04761-x)
Supplement: Supplementary file 3 — Description of Additional Supplementary Files [file 42003_2023_4761_MOESM3_ESM.pdf]

Description of Additional Supplementary Files

File name: supplementary_data1.txt

Description: The source data behind Figure 4b

File name: supplementary_data2.txt

Description: The source data behind Figure 5b

File name: supplementary_data3.txt

Description: The source data behind Figure 6a

File name: supplementary_data4.txt

Description: The source data behind Figure 6b
