# Supplementary material for: SpaDecon: cell-type deconvolution in spatial transcriptomics with semi-supervised learning
Source: Commun Biol. 2023 Apr 7;6:378. doi: 10.1038/s42003-023-04761-x (PMC10082183; doi:10.1038/s42003-023-04761-x)
Supplement: Supplementary file 8 — Reporting Summary [file 42003_2023_4761_MOESM8_ESM.pdf]

nature portfolio

Corresponding author(s): Mingyao Li, Kyle Coleman

Last updated by author(s): 02/09/2023

Reporting Summary

Nature Portfolio wishes to improve the reproducibility of the work that we publish. This form provides structure for consistency and transparency in reporting. For further information on Nature Portfolio policies, see our Editorial Policies and the Editorial Policy Checklist.

Statistics

For all statistical analyses, confirm that the following items are present in the figure legend, table legend, main text, or Methods section.

n/a	Confirmed	
☐	☒	The exact sample size (*n*) for each experimental group/condition, given as a discrete number and unit of measurement
☐	☒	A statement on whether measurements were taken from distinct samples or whether the same sample was measured repeatedly
☐	☒	The statistical test(s) used AND whether they are one- or two-sided<br>*Only common tests should be described solely by name; describe more complex techniques in the Methods section.*
☐	☒	A description of all covariates tested
☒	☐	A description of any assumptions or corrections, such as tests of normality and adjustment for multiple comparisons
☒	☐	A full description of the statistical parameters including central tendency (e.g. means) or other basic estimates (e.g. regression coefficient) AND variation (e.g. standard deviation) or associated estimates of uncertainty (e.g. confidence intervals)
☒	☐	For null hypothesis testing, the test statistic (e.g. *F*, *t*, *r*) with confidence intervals, effect sizes, degrees of freedom and *P* value noted<br>*Give P values as exact values whenever suitable.*
☒	☐	For Bayesian analysis, information on the choice of priors and Markov chain Monte Carlo settings
☒	☐	For hierarchical and complex designs, identification of the appropriate level for tests and full reporting of outcomes
☒	☐	Estimates of effect sizes (e.g. Cohen's *d*, Pearson's *r*), indicating how they were calculated

Our web collection on statistics for biologists contains articles on many of the points above.

Software and code

Policy information about availability of computer code

Data collection	No software was used for data collection.
Data analysis	SpaDecon v1.1.1 (https://github.com/kpcoleman/SpaDecon), RCTD v1.2.0 (https://github.com/dmcable/spacexr), SPOTlight v0.1.7 (https://github.com/MarcElosua/SPOTlight), Stereoscope v0.2.0 (https://github.com/almaan/stereoscope), Cell2location v0.1 (https://github.com/BayraktarLab/cell2location), and MuSiC v0.2.0 (https://github.com/xuranw/MuSiC) were used for cell-type deconvolution. Scanpy v1.8.2 was used for data preprocessing.

For manuscripts utilizing custom algorithms or software that are central to the research but not yet described in published literature, software must be made available to editors and reviewers. We strongly encourage code deposition in a community repository (e.g. GitHub). See the Nature Portfolio guidelines for submitting code & software for further information.

Data

Policy information about availability of data

All manuscripts must include a data availability statement. This statement should provide the following information, where applicable:

- Accession codes, unique identifiers, or web links for publicly available datasets
- A description of any restrictions on data availability
- For clinical datasets or third party data, please ensure that the statement adheres to our policy

We analyzed two Visium datasets and two ST datasets, each of which required an annotated scRNA-seq dataset. These data are publicly available and can be obtained through the following websites or GEO accession numbers:
(1) Mouse Brain (SRT: https://support.10xgenomics.com/spatial-gene-expression/datasets/1.1.0/V1_Mouse_Brain_Sagittal_Anterior; scRNA-seq: https://portal.brain-map.org/atlases-and-data/rnaseq/mouse-whole-cortex-and-hippocampus-smart-seq); scRNA-seq: GSE71585;

(2) Breast Cancer (SRT: https://support.10xgenomics.com/spatial-gene-expression/datasets/1.1.0/V1_Breast_Cancer_Block_A_Section_1; scRNA-seq: GSE75688);
(3) Melanoma (SRT: https://www.spatialresearch.org/resources-published-datasets/doi-10-1158-0008-5472-can-18-0747/; scRNA-seq: GSE72056);
(4) Pancreatic Ductal Adenocarcinoma (SRT: GSM3405534; scRNA-seq: GSE111672)

Field-specific reporting

Please select the one below that is the best fit for your research. If you are not sure, read the appropriate sections before making your selection.

☒ Life sciences ☐ Behavioural & social sciences ☐ Ecological, evolutionary & environmental sciences

For a reference copy of the document with all sections, see nature.com/documents/nr-reporting-summary-flat.pdf

Life sciences study design

All studies must disclose on these points even when the disclosure is negative.

Sample size	Our study did not involve data collection. For each of the four spatially resolved transcriptomics datasets we analyzed, there was a predefined number of spots with measured gene expression. We utilized all spots when analyzing a given tissue section. We did not use statistical methods to determine sample sizes.
Data exclusions	For two of the tissue sections we analyzed (10X Visium mouse brain and 10X Visium breast cancer), we used subsets of the single-cell reference datasets due to the inability of competing methods to use the full reference datasets.
Replication	We did not replicate our results, as we instead compared them with known biological properties and evaluated performance through benchmark evaluations.
Randomization	This is not relevant to our study as all tissue sections were analyzed separately.
Blinding	This is not relevant as our study did not involve data collection.

Reporting for specific materials, systems and methods

We require information from authors about some types of materials, experimental systems and methods used in many studies. Here, indicate whether each material, system or method listed is relevant to your study. If you are not sure if a list item applies to your research, read the appropriate section before selecting a response.

Materials & experimental systems

n/a	Involved in the study
☒	☐ Antibodies
☒	☐ Eukaryotic cell lines
☒	☐ Palaeontology and archaeology
☒	☐ Animals and other organisms
☒	☐ Human research participants
☒	☐ Clinical data
☒	☐ Dual use research of concern

Methods

n/a	Involved in the study
☒	☐ ChIP-seq
☒	☐ Flow cytometry
☒	☐ MRI-based neuroimaging
