# Supplementary material for: SpaDecon: cell-type deconvolution in spatial transcriptomics with semi-supervised learning
Source: Commun Biol. 2023 Apr 7;6:378. doi: 10.1038/s42003-023-04761-x (PMC10082183; doi:10.1038/s42003-023-04761-x)
Supplement: Supplementary file 1 — Li_Peer Review File [file 42003_2023_4761_MOESM1_ESM.pdf]

nature portfolio

Peer Review File

SpaDecon: cell-type deconvolution in spatial transcriptomics with semi-supervised learning

Open Access This file is licensed under a Creative Commons Attribution 4.0 International License, which permits use, sharing, adaptation, distribution and reproduction in any medium or format, as long as you give appropriate credit to the original author(s) and the source, provide a link to the Creative Commons license, and indicate if changes were made. In the cases where the authors are anonymous, such as is the case for the reports of anonymous peer reviewers, author attribution should be to 'Anonymous Referee' followed by a clear attribution to the source work. The images or other third party material in this file are included in the article's Creative Commons license, unless indicated otherwise in a credit line to the material. If material is not included in the article's Creative Commons license and your intended use is not permitted by statutory regulation or exceeds the permitted use, you will need to obtain permission directly from the copyright holder. To view a copy of this license, visit http://creativecommons.org/licenses/by/4.0/.

This manuscript has been previously reviewed at another Springer Nature journal. This document only contains reviewer comments and rebuttal letters for versions considered at Communications Biology.

Reviewers' comments:

Reviewer #1 (Remarks to the Author):

Coleman et al. proposed a new method “SpaDecon” for deconvolving SRT data in a semi-supervised approach. They first use H&E image to smooth the data, then co-embed spots and single-cells in the same low dimensional space, take the centroid of cell type j, and compute the distance between spot i and the centroid of cell type j as the proportion of cell type j at spot i.

The results are encouraging but we have major concerns about the assumptions, the clarity of description and the choice of methods for benchmarking. Our major concerns are below.

1. The assumption that the distance between spot i and the centroid of cell type j models the proportion of cell type j at spot i is not intuitive and sounds arbitrary. The authors need to provide a convincing argument for this assumption.

2. “Specifically, for a given spot, the benchmark cell-type distribution is the mean of the cell-type proportions estimated by SpaDecon, RCTD, SPOTlight, and Stereoscope for that spot.” – this part of the methodology is also questionable. All methods may fail to detect a rare cell type or a finer-resolution subtype and/or can give a wide variation in prediction. Thus, the mean may not reflect the true cell type fraction.

3. The descriptions in the Methods section in general and particularly in the sub-section on “Construction of adjacency matrix” are very operational/algorithmic but need to provide more intuition. Along the same lines, Figure 1 needs a lot of improvement – as presented, it is not successfully conveying the complex ideas.

4. “A major drawback of these cell-type deconvolution methods is that they do not take advantage of the high-resolution histology image and the spatial locations of the spots for which gene expression levels are measured.” – this statement is true about the chosen methods, but cell2location (PMID: 35027729) addresses these concerns and needs to be included in the benchmarking.

5. The authors also need to benchmark SpaDecon against cell2location for the cases where cell2location showed successful mapping of rare celltypes and fine-grained subtypes.

Point-by-Point Response to Reviews

We sincerely thank the reviewer for their constructive comments, which have helped improve the quality of our manuscript. We have made substantial improvements to our manuscript and highlighted the revised/new parts in red for ease of reviewing. Below are our point-by-point responses to the reviewer' comments. The original reviewers' comments are in **bold** *italics* and our responses are in normal font colored in blue.

Reviewer #1 (Remarks to the Author):

Coleman et al. proposed a new method "SpaDecon" for deconvolving SRT data in a semi-supervised approach. They first use H&E image to smooth the data, then co-embed spots and single-cells in the same low dimensional space, take the centroid of cell type j, and compute the distance between spot i and the centroid of cell type j as the proportion of cell type j at spot i.

The results are encouraging but we have major concerns about the assumptions, the clarity of description and the choice of methods for benchmarking. Our major concerns are below.

1. The assumption that the distance between spot i and the centroid of cell type j models the proportion of cell type j at spot i is not intuitive and sounds arbitrary. The authors need to provide a convincing argument for this assumption.

We apologize for the lack of clarity. Our estimation procedure for the cell type proportions was inspired by the Deep Embedded Clustering (DEC) method (https://arxiv.org/abs/1511.06335), which simultaneously learns feature representations and cluster assignments using deep neural networks. In DEC, they first perform dimension reduction using an autoencoder and then use the Student's t-distribution as a kernel to measure the similarity between an embedded data point z_i and a centroid μ_j, where the similarity q_{ij} between z_i and μ_j is defined as,

$$q_{ij} = \frac{\left(1 + \|z_i - \mu_j\|^2\right)^{-1}}{\sum_{j'}\left(1 + \|z_i - \mu_{j'}\|^2\right)^{-1}}.$$

In the DEC paper, q_{ij} is interpreted as the probability of assigning data point i to cluster j (i.e., a soft assignment). In SpaDecon, we obtain the clustering centroid μ_j through supervised clustering analysis on the scRNA-seq reference where cell type annotation information is known. Therefore, each centroid corresponds to the multi-dimensional average of a cell type. Following the DEC paper, for a given spot i, we interpret the similarity q_{ij} as the probability of assigning the spot to cell type j, which is an approximate of the proportion of cells in spot i belong to cell type j.

We also note that our approach shares similarity with DSTG (PMCID: PMC8425268), which calculates the relative distances of spots to pseudo-spots of known composition, but in SpaDecon, we use the centroids of purely one cell type when calculating the cell type proportions.

We have added the above explanation in the Methods section of the revised manuscript, which is also pasted below:

"Following the Deep Embedded Clustering paper [27], q_{ij} is interpreted as the probability of assigning spot i to cell type j, which is an approximate of the proportion of cells in spot i belong to cell type j. Similar cell type proportion estimation procedure has been utilized by DSTG [28], which calculates the relative distances of spots to pseudo-spots of known composition, but in SpaDecon, we use the centroids of purely one cell type as the centroid when estimating the cell type proportions."

2. "Specifically, for a given spot, the benchmark cell-type distribution is the mean of the cell-type proportions estimated by SpaDecon, RCTD, SPOTlight, and Stereoscope for that spot." – this part of the methodology is also questionable. All methods may fail to detect a rare cell type or a finer-resolution subtype and/or can give a wide variation in prediction. Thus, the mean may not reflect the true cell type fraction.

Our initial motivation of using the mean cell type proportions estimated across different methods was to avoid favoring a particular method, but we agree with the reviewer that taking the average may fail to evaluate the performance of different methods in detecting a rare cell type. To address this potential concern, we conducted new benchmarking simulations. For each simulation, we identified the two cell types with the lowest mean estimated proportions across all methods when evaluated on the real SRT dataset. For each of these cell types, we set the benchmark proportion equal to the minimum, instead of the mean, estimated proportion across all methods for each spot. The benchmark proportions for all other cell types were set equal to the mean of estimated proportions across all methods, and the proportions for each spot were normalized to sum to 1. We have updated the benchmarking analyses using this updated simulation scheme in the revised manuscript.

3. The descriptions in the Methods section in general and particularly in the sub-section on "Construction of adjacency matrix" are very operational/algorithmic but need to provide more intuition. Along the same lines, Figure 1 needs a lot of improvement – as presented, it is not successfully conveying the complex ideas.

We have included more details regarding the intuition of constructing and utilizing an adjacency matrix in the Results section "Overview of SpaDecon and evaluation". The added sentences are also included here:

"Because the number of cells per spot is small (~1 to 30) [2], the observed expression at each spot is noisy. However, spots that are physically close and share similar histological features are expected to have similar gene expression and cell-type proportions, which implies that the gene expression for each spot can be denoised by aggregating information from its neighboring spots. This motivated us to use a graph-based local smoothing approach to denoise the observed gene expression."

Following your suggestion, we also revised Figure 1. We hope this new version now conveys our idea clearly.

4. "A major drawback of these cell-type deconvolution methods is that they do not take advantage of the high-resolution histology image and the spatial locations of the spots for which gene expression levels are measured." – this statement is true about the chosen methods, but cell2location (PMID: 35027729) addresses these concerns and needs to be included in the benchmarking.

Cell2location does not directly utilize spatial location or histology information when inferring cell-type proportions. This method requires a user-selected hyperparameter for the number of cells per gene capture location, which is not generally known. To estimate this hyperparameter, the authors recommend the user visually inspect the histology image or use a cell segmentation algorithm to count the approximate number of cells per gene capture location.

5. The authors also need to benchmark SpaDecon against cell2location for the cases where cell2location showed successful mapping of rare celltypes and fine-grained subtypes.

Following your suggestion, we have included cell2location in all analyses. Based on visual examination and prior knowledge of the general locations of cell types in the real SRT datasets, the SpaDecon estimated proportions were found to be more accurate than cell2location. We also quantitatively demonstrated the superior performance of SpaDecon through evaluation metrics calculated using proportions estimated for pseudo-SRT datasets. For a vast majority of cell types and spots, the SpaDecon estimated proportions were shown to have lower MSE and JSD with the benchmark proportions when compared with cell2location. One major drawback that we have noticed about cell2location is its computational speed. Cell2location requires a GPU to run in a reasonable amount of time and, even with a GPU, had a mean run time of over an hour for one single tissue section. The time to train the cell2location model on a CPU can be more than 50 hours. The slow speed will make it impractical to use cell2locatio in large-scale studies.

Reviewers' comments:

Reviewer #1 (Remarks to the Author):

We thank the authors for their attempts to address our previous comments and concerns. However, some major concerns still remain, as we describe below.

We understand that the pseudo-SRT dataset was generated to mimic a real SRT dataset. The authors have also included steps to address our previous concerns and explain the process better. With a clearer understanding of this method, we are now concerned about the soundness of this part of the paper. The authors need to appreciate that, since this process is still dependent on the "benchmark cell-type proportions" estimated by the methods being benchmarked, the methods are not truly blind about the pseudo-SRT dataset.

A simpler approach to take single-cell resolution ST data like MERFISH and then imposing Visium-sized spots on it should have worked and would give a ground truth that is free from model estimation.

Furthermore, it is unclear why the authors choose only two cell types as "rare" in the first pseudo-SRT dataset. Similarly, why were macrophages and tuft cells were chosen as rare for that dataset? For the other datasets, we could not find the details of the above steps.

The authors demonstrated their algorithm to be faster than other methods. This result needs an explanation. Which part(s) of their algorithm is saving the execution time compared to the other methods? We understand that different methods have different principles, so a direct comparison might not be possible. Nevertheless, some algorithmic intuition can be offered. For example, the Bayesian optimization of cell2location is probably making the run time longer. It is also easy to comment on the number of iterations/epochs required by the methods and the bottleneck step of each method. The title of Fig 7 should not be time complexity. "Time complexity" refers to an established method to analytically represent how an algorithm's runtime scales with increasing the size of the input data.

Finally, overlaying the color-scaled spots on H&E images actually makes the images less readable. However, we will leave this point to the Editors' discretion.

Point-by-Point Response to Reviews

We sincerely thank the reviewer for their constructive comments, which have helped improve the quality of our manuscript. We have revised our manuscript and highlighted the revised/new parts in red for ease of reviewing. Below are our point-by-point responses to the reviewer' comments. The original reviewer' comments are in **bold** *italics* and our responses are in normal font colored in blue.

Reviewer #1 (Remarks to the Author):

We thank the authors for their attempts to address our previous comments and concerns. However, some major concerns still remain, as we describe below.

1. We understand that the pseudo-SRT dataset was generated to mimic a real SRT dataset. The authors have also included steps to address our previous concerns and explain the process better. With a clearer understanding of this method, we are now concerned about the soundness of this part of the paper. The authors need to appreciate that, since this process is still dependent on the "benchmark cell-type proportions" estimated by the methods being benchmarked, the methods are not truly blind about the pseudo-SRT dataset.

We appreciate the reviewer's thoughtful comment on benchmark evaluation using pseudo-ST data. When constructing the pseudo-SRT dataset, we chose to calculate benchmark proportions using estimated cell-type proportions, as opposed to random proportions, so that the resulting pseudo-spots would be composed of realistic distributions of cell types. If we sampled according to the estimated proportions of one method, the benchmark evaluations would clearly be biased towards that method and others that inherently generate cell-type proportion estimates following that distribution. Since we used the mean of the estimates across all methods, however, the resulting distribution of proportions will not inherently match the distribution of proportion estimates of any particular method. As a result, even though we use the results of the methods to generate the pseudo-SRT dataset, the evaluations are not biased towards any one method over the others.

2. A simpler approach to take single-cell resolution ST data like MERFISH and then imposing Visium-sized spots on it should have worked and would give a ground truth that is free from model estimation.

We thank the reviewer for this constructive suggestion. We conducted additional benchmark evaluations following what you suggested. However, we have noticed a few problems with this approach, which might have been overlooked by others. First, cells in a MERFISH dataset are identified using cell segmentation methods which are incapable of isolating all cells in a tissue section, particularly in regions with dense cell populations. In fact, we have been analyzing a large-scale MERFISH dataset through an independent project, and one challenge that we encountered in that project is how to reliably segment cells from tissue regions that have high cell density. Due to complications like this, a dataset constructed by imposing Visium-sized spots on MERFISH data will encompass fewer cells per spot than would occur in a real Visium dataset. In order to construct pseudo-Visium spots with realistic numbers of cells, a larger spot diameter would be required. However, this would then have the potential for pseudo-spots to contain cell types that are generally not close enough to be located in the same spots of a real Visium dataset. We are aware that several other cell-type deconvolution papers evaluated the performance of their methods using MERFISH or seqFISH-based pseudo-SRT data, however, none of these papers discussed the potential issue caused by cell segmentation.

Another potential problem with MERFISH-based pseudo-ST data is that cells segmented in a MERFISH experiment are not annotated, meaning that a label transfer method and reference scRNA-seq dataset are required to assign each cell to a cell type. Thus, the benchmark cell-type proportions depend heavily on the label transfer method and reference dataset utilized. To illustrate this fact, we analyzed a MERSCOPE dataset generated from mouse brain by Vizgen (https://info.vizgen.com/mouse-brain-map?submissionGuid=617906b7-e3be-4cba-a873-441336a3d7b9). We performed two sets of benchmark evaluations, where in each set we implemented a different label transfer method to annotate the MERSCOPE dataset. Other than the label transfer method, the processes for constructing the pseudo-Visium datasets were identical.

First, each segmented cell was assigned a cell-type label using SingleR (Aran et al. 2019 Nature Immunology, https://github.com/dviraran/SingleR) and ItClust (Hu et al. 2020 Nature Machine Intelligence, https://github.com/jianhuupenn/ItClust), two leading single-cell label transfer methods. For both methods, the mouse whole cortex and hippocampus SMART-seq dataset from the Allen Brain Institute (described in the manuscript) was used as the reference dataset for cell-type annotation. We then imposed Visium-sized pseudo-spots of size 55µmx55µm on the MERSCOPE dataset and summed the gene counts of all cells within the same pseudo-spot to obtain spot-level gene expression features.

Two sets of benchmark cell-type proportions were then calculated using each of the label transfer results. Cell-type deconvolution was performed by each method on the resulting pseudo-Visium dataset and estimated proportions were compared with the ItClust and SingleR generated benchmark proportions separately.

The following two plots show the cell-type level MSE and spot-level JSD between the benchmark and estimated proportions for each method, where the benchmark proportions were determined using SingleR as the method for cell-type annotation.

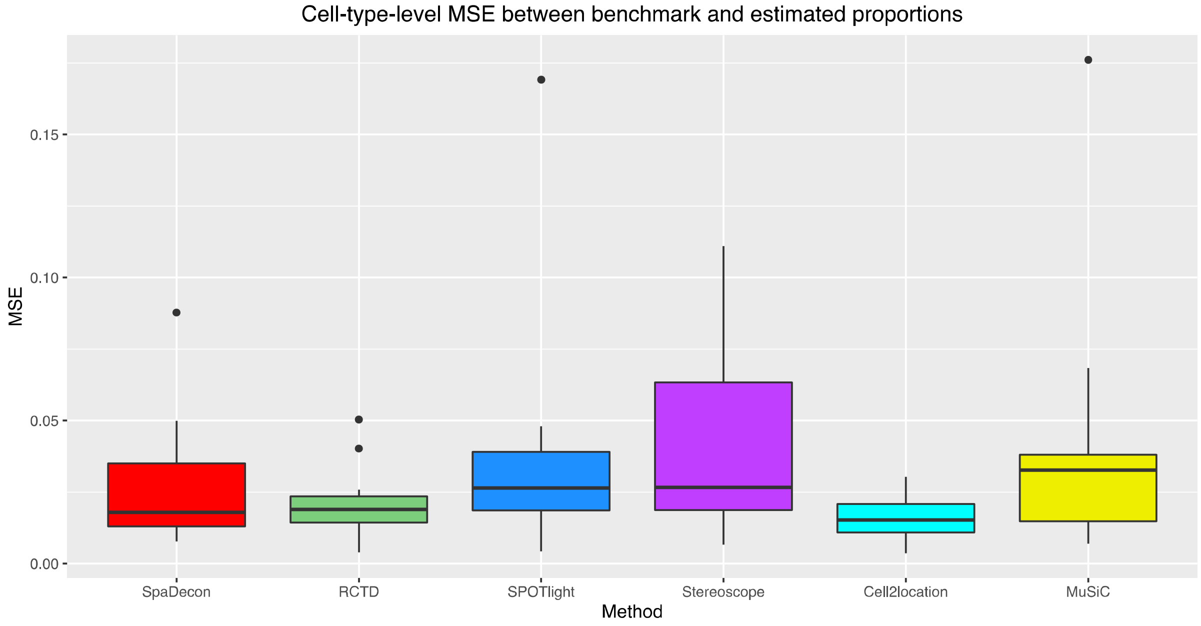

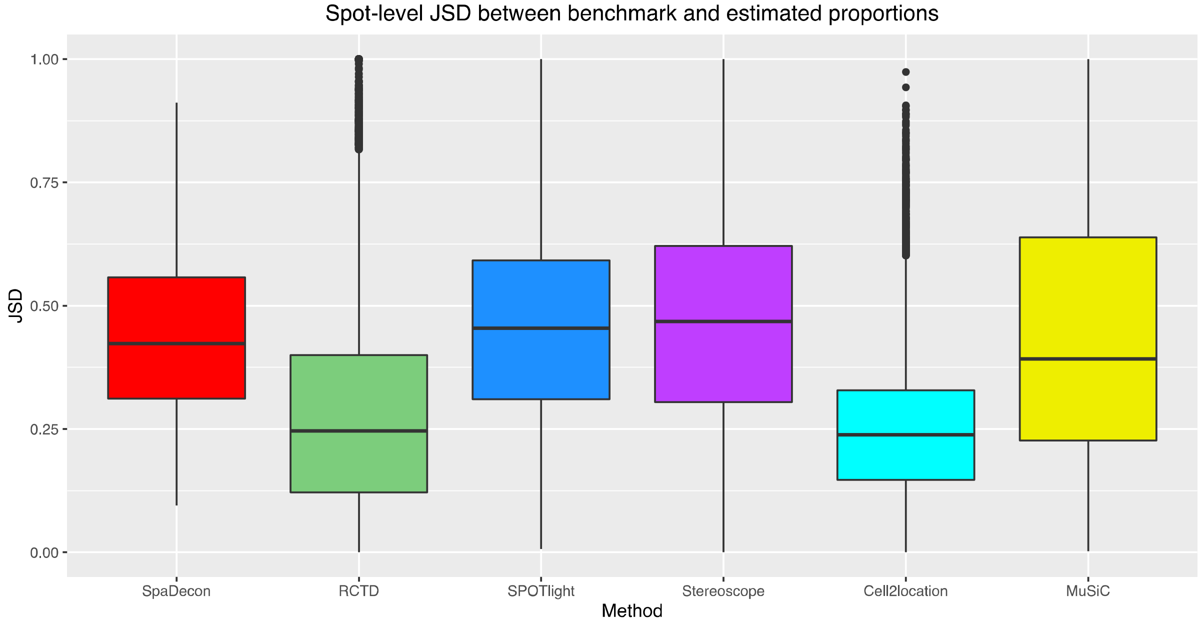

The next two plots show the cell-type level MSE and spot-level JSD between the ItClust-generated benchmark and estimated proportions for each method.

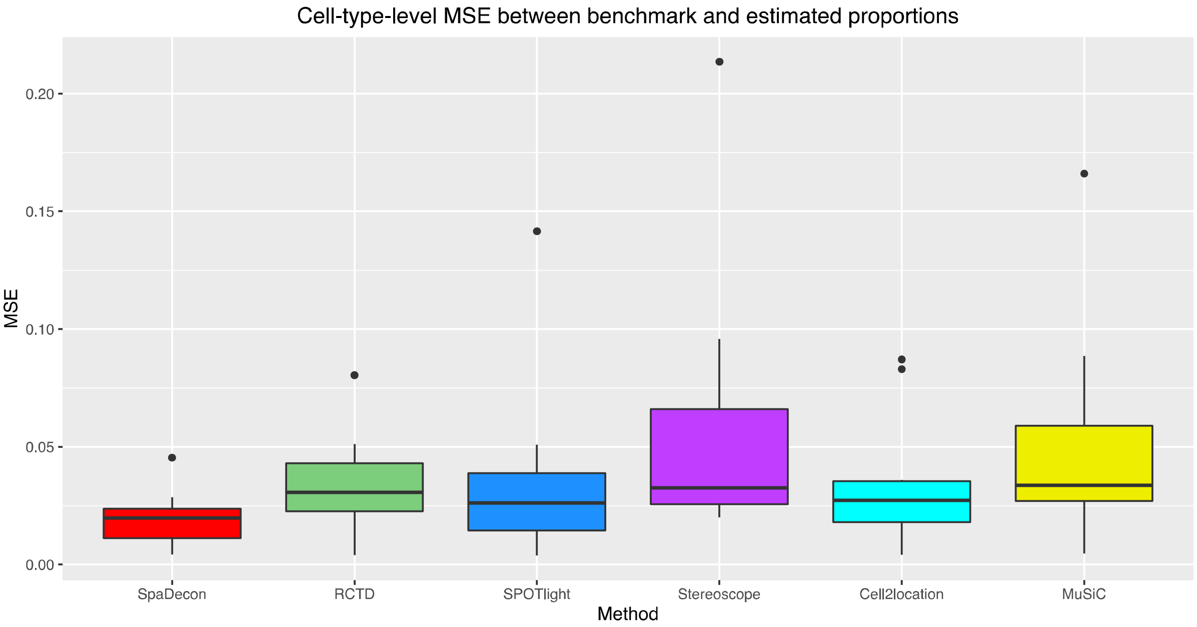

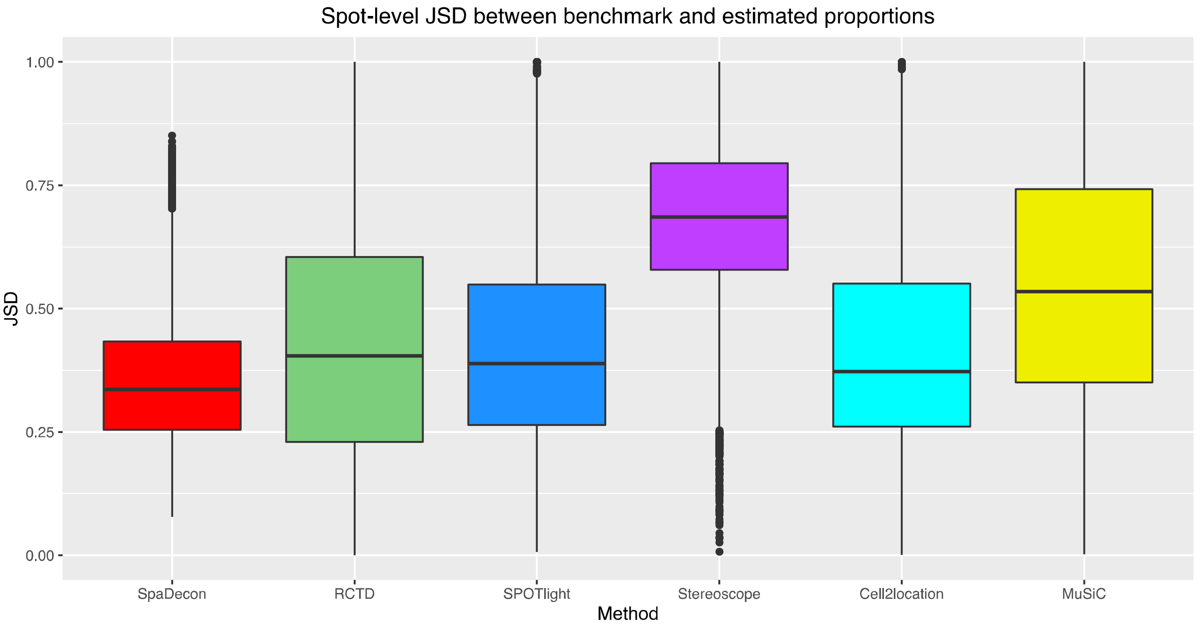

As evidenced by the above plots, performance on a pseudo-Visium dataset constructed using MERSCOPE data is heavily influenced by the cell-type annotation method. Since there is no definitive way to determine the most accurate label transfer method for this situation, we cannot be confident enough in the MERSCOPE cell-type assignments to ensure the validity of these evaluations. To this point, we argue that our original benchmark evaluations provide a more reliable summary of method performance on realistic pseudo-SRT datasets. Although our original approach has the potential problem that the reviewer pointed out, we think the amount of information leaked to each individual method is small because we used the average cell type proportion from all methods. If it is preferable, we can include our MERSCOPE benchmark evaluations in the supplement.

As we are concerned with MERFISH-based pseudo-ST data, we discussed this topic with one of the developers of the MERFISH technology (personal communication). The developer agreed that the choice of label transfer method and scRNA-seq dataset can dramatically influence the results. As a result, they stated that the most accurate way to annotate cells is by individually investigating the marker gene expression levels of each cell segmented in the MERFISH experiment, which is a costly and time-consuming process.

3. Furthermore, it is unclear why the authors choose only two cell types as "rare" in the first pseudo-SRT dataset. Similarly, why were macrophages and tuft cells were chosen as rare for that dataset? For the other datasets, we could not find the details of the above steps.

For all pseudo-SRT datasets we generated, we chose two rare cell types based on the estimated proportions of the cell-type deconvolution methods. The rare cell types were chosen as the two cell types with the lowest mean estimated proportions across each of the methods. We incorporated this step to address the reviewer's concern of not evaluating the ability of the methods to identify rare cell types. Below we show the distributions of the benchmark cell-type proportions of rare cell types for each pseudo-SRT dataset we generated. In each dataset, the median cell type proportions for the two rare cell types are less than 5%, which are similar to rare cell types seen in real studies.

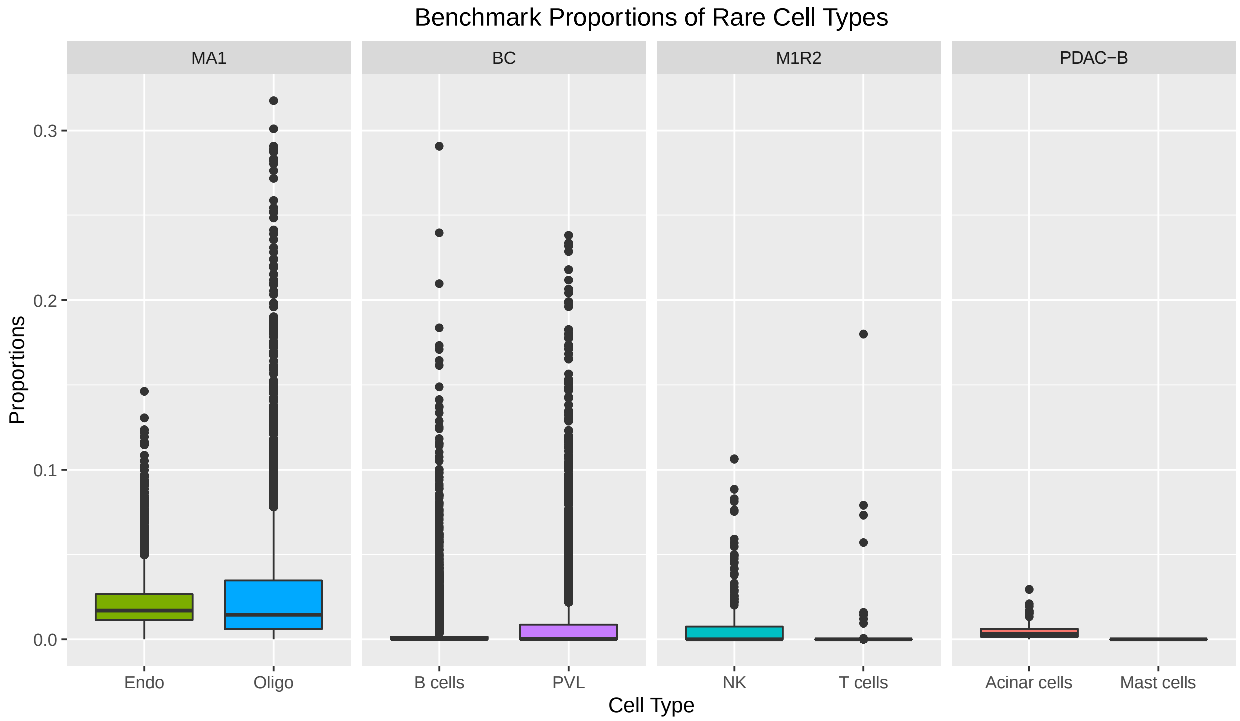

4. The authors demonstrated their algorithm to be faster than other methods. This result needs an explanation. Which part(s) of their algorithm is saving the execution time compared to the other methods? We understand that different methods have different principles, so a direct comparison might not be possible. Nevertheless, some algorithmic intuition can be offered. For example, the Bayesian optimization of cell2location is probably making the run time longer. It is also easy to comment on the number of iterations/epochs required by the methods and the bottleneck step of each method.

SpaDecon's impressive runtime can be credited, in part, to the method's approach for dimensionality reduction of the SRT gene expression data. As detailed in the methods section of the paper, SpaDecon performs highly variable gene selection and, importantly, implements a stacked autoencoder to greatly reduce the dimensionality of the data to 32-128 features per spot while maintaining the between-spot heterogeneity. The stacked autoencoder is pre-trained layer by layer, which enables us to fine-tune the entire network for a fewer number of epochs, as opposed to fully training the network with random parameter initializations. This dimensionality reduction leads to a faster runtime for the supervised version of the algorithm, in which cluster centroids are optimized in the latent feature space using the annotated scRNA-seq data. We have added this explanation to the Discussion section of the paper.

5. The title of Fig 7 should not be time complexity. "Time complexity" refers to an established method to analytically represent how an algorithm's runtime scales with increasing the size of the input data.

Thank you for this comment. We have changed the title of Fig 7 to “Runtime”.

6. Finally, overlaying the color-scaled spots on H&E images actually makes the images less readable. However, we will leave this point to the Editors' discretion.

We have explored different ways of visualizing the deconvolution results, including the one that the reviewer suggested. However, we found it difficult to interpret the plots in the absence of histology images. Based on our own investigations, we found overlaying the color-scaled spots on H&E images offers the best visual clarity and overall information about the results. If the editor agrees that this makes the images less readable, we will remove the histology from the background of the plots.

REVIEWERS' COMMENTS:

Reviewer #1 (Remarks to the Author):

The authors have adequately addressed our suggestions and comments. Some of these are valuable discussion points and beneficial for the community.
